# Interpreting linear support vector machine models with heat map molecule coloring

**DOI:** 10.1186/1758-2946-3-11

**Published:** 2011-03-25

**Authors:** Lars Rosenbaum, Georg Hinselmann, Andreas Jahn, Andreas Zell

**Affiliations:** 1 University of Tübingen, Center for Bioinformatics (ZBIT), Sand 1, 72076 Tübingen, Germany

## Abstract

**Background:**

Model-based virtual screening plays an important role in the early drug discovery stage. The outcomes of high-throughput screenings are a valuable source for machine learning algorithms to infer such models. Besides a strong performance, the interpretability of a machine learning model is a desired property to guide the optimization of a compound in later drug discovery stages. Linear support vector machines showed to have a convincing performance on large-scale data sets. The goal of this study is to present a heat map molecule coloring technique to interpret linear support vector machine models. Based on the weights of a linear model, the visualization approach colors each atom and bond of a compound according to its importance for activity.

**Results:**

We evaluated our approach on a toxicity data set, a chromosome aberration data set, and the maximum unbiased validation data sets. The experiments show that our method sensibly visualizes structure-property and structure-activity relationships of a linear support vector machine model. The coloring of ligands in the binding pocket of several crystal structures of a maximum unbiased validation data set target indicates that our approach assists to determine the correct ligand orientation in the binding pocket. Additionally, the heat map coloring enables the identification of substructures important for the binding of an inhibitor.

**Conclusions:**

In combination with heat map coloring, linear support vector machine models can help to guide the modification of a compound in later stages of drug discovery. Particularly substructures identified as important by our method might be a starting point for optimization of a lead compound. The heat map coloring should be considered as complementary to structure based modeling approaches. As such, it helps to get a better understanding of the binding mode of an inhibitor.

## Background

High-throughput screenings (HTS) play an important role in the early drug discovery stage. The data of these HTS are a valuable, but challenging resource for machine learning algorithms to infer predictive structure-activity relationship models for virtual screening [1]. In later stages of drug discovery, a lead compound is optimized for desired biophysical properties. However, as a lead compound becomes increasingly tailored to a target, there is generally less tolerance for introducing changes without an intrinsic affinity penalty [2]. Thus, besides a strong performance, the reasons that lead to a prediction of a compound as active or inactive is important for a medicinal chemist in lead optimization.

Recent examples of interpretable methods applied to cheminformatic problems include Naïve Bayes, decision trees, and k-nearest neighbor approaches. Bender et al. [3] applied Bayesian learning to radial atom environments and used the information gain to assess the significance of a substructure. Han et al. [4] trained decision trees on several PubChem HTS data sets. Swamidass et al. [5] introduced the Influence Relevance Voter, an interpretable method based on a supervised artificial neural network in combination with a k-nearest neighbor approach. Recently, Mohr et al. [6] employed a potential support vector machine (SVM) in combination with a maximum-common subgraph kernel to predict the genotoxicity of a compound.

Using a two step procedure, Mohr et al. labeled the atoms of a compound as important or unimportant for genotoxicity. First, the design of the potential SVM allows for the assignment of weights to atoms. Second, based on the weights, an atom is classified as important for genotoxicity if a predefined threshold is exceeded.

Linear SVMs in combination with sparse molecular fingerprints showed a convincing performance on several large-scale data sets [7]. In contrast to their nonlinear counterpart, linear SVMs are no black box concerning interpretability because they do not perform a nonlinear mapping from the input space to a high-dimensional feature space. Linear SVMs learn a linear discriminant function, which assigns a weight to each fingerprint feature of the input space. Recent studies [8,9] indicate that the interpretation of linear SVM models is possible for small regression data sets. Both approaches exploited the weights of a linear support vector regression model to extract patterns which are important for activity or selectivity against a certain protein target.

The aim of this study is to present a visualization method that allows for the interpretation of linear SVM models of large-scale data sets. We use the weights of the linear discriminant function to assign a score to each atom or bond of a compound. Based on these scores, a color is assigned to each atom or bond of a compound. We tested the visualization approach on the Kazius Ames toxicity data set [10], the chromosome aberration data set compiled by Mohr et al. [6], and the maximum unbiased validation data sets [11].

The results show that our method is able to sensibly visualize the structure-property and structure-activity information of a linear SVM model. The heat map visualization can be combined with structure based modeling approaches to gain a better understanding of the binding mode of a compound and therefore help medicinal chemists in lead optimization.

## Methods

### Nonlinear vs. linear SVM models

A virtual screening data set of *l *compounds can be represented as a set of *l *labeled fingerprints of compounds (**x***_i_*, *y_i_*), *i *= 1, ..., *l*, **x***_i _*= (*x*_*i*1_, ..., *x_im_*), *x_ij _*∈ ℝ, *y_i _*∈ {-1, +1}. In the case of binary substructure fingerprints, each *x_ij _*∈ {0, 1} is an indicator for the presence or absence of a pattern (or fingerprint feature) *j *in compound *i*. Every compound is labeled either as active (*y_i _*= +1) or inactive (*y_i _*= -1). SVMs learn a discriminant function of the form(1)

where *SV *= {**x***_i_*|*α_i _*> 0} is the set of support vectors, *k *is the kernel function and *ϕ *is a mapping from the input space to a high-dimensional feature space. In general, the actual mapping *ϕ *is unknown. The kernel *k *implicitly performs the mapping and the calculation of the dot product *ϕ *(**x***_i_*)*^T ^ϕ *(**x**). Thus, in case of nonlinear SVMs, it is impossible to assess how a certain training set feature *x_ij _*contributes to the kernel similarity *k*(**x***_i _*, **x**). Hence, nonlinear SVMs are a black box concerning interpretability.

In case of linear SVMs, the mapping *ϕ *is the identity, which results in a linear discriminant function of the form(2)

where the weight vector **w **= (*w*_1_, ..., *w_m_*) is optimized such that the separating hyperplane defined by *f*(**x**) has maximum margin. The discriminant function is employed to predict the class *sign*(*f*(**x**)) of an unknown sample. The value of *f*(**x**) is called prediction value. Compounds with a prediction value close to zero are close to the separating hyperplane. Consequently, the classification can be interpreted as less certain.

The weight vector **w **can be expressed by(3)

Hence, the weight vector **w **contains the weighted features of the support vectors. The SVM assigns an *α *> 0 if the compound is necessary for class separation. Thus, a compound *i *which contains no information for class separation will have *α_i _*= 0. Its features *x_ij _*will not have a weight *w_j _*≠ 0 unless another compound with an *α *> 0 also contains the pattern. Additionally, the weight *w_ij _*= *α_i_y_i_x_ij _*of a feature of a compound with *α_i _*> 0 is positive if the class of a compound **x***_i _*is labeled active (*y_i _*= +1) and negative otherwise.

The linear discriminant function *f*(**x**) in combination with binary substructure fingerprints is equivalent to the Free-Wilson formulation in chemometrics [12,13]. The Free-Wilson formulation assigns each pattern *p_j _*a weight *w_j _*according to its contribution to activity. In contrast to the Free-Wilson formulation, a linear SVM model is a classification model and not a regression model. Thus, the weight of a feature does not represent the actual contribution to binding affinity, but the relative importance of a feature. Still, a linear SVM model can in principle be interpreted in the same way as a Free-Wilson model. A pattern with large positive weight is expected to be important for activity of a compound while a pattern with large negative weight should represent inactive or non relevant parts of a molecule. A pattern with a weight close to zero should be unimportant for class separation.

A more detailed description of maximum-margin based classifiers can be found in Schölkopf and Smola [14] for further reading.

### Molecular Encodings

For structure-based classifiers, it is common to encode the molecular graph of a compound with binary fingerprints for large-scale learning tasks in cheminformatics. Each bit of a fingerprint indicates the presence or absence of a fingerprint feature. The specific choice of molecular encoding is crucial to obtain an interpretable linear model. The employed encoding must ensure that the set of atoms or bonds which a fingerprint feature represents is available while calculating the molecular encoding. Consequently, the weight of a fingerprint feature can be mapped back to those atoms or bonds. The mapping from sets of atoms or bonds to fingerprint features needs not to be injective. If a collision occurs, the weight of a feature is mapped to all sets of atoms or bonds that caused the collision. Common molecular encodings that generate interpretable features are radial atom environments [3], depth first search paths [15], or extended connectivity fingerprints (ECFP) [16].

**Figure 1:**
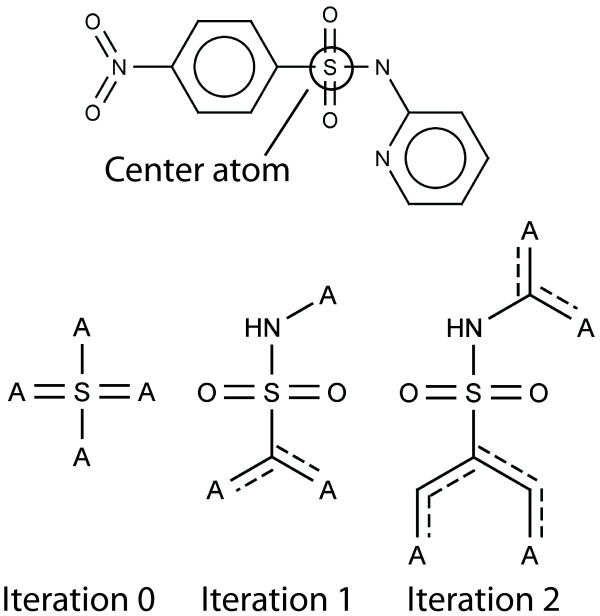
**Illustration of the ECFP**. Each circular substructure around a center atom represents an ECFP feature. The circular substructure is grown in each iteration at the attachment points A. Any atom can be matched on an attachment point. Aromatic bonds are marked by a dashed line.

We employed a variant of the ECFP to encode the molecules because ECFP features are intuitively interpretable and can be mapped back to the topology of a chemical graph. Each ECFP feature of a fingerprinted compound represents a circular substructure around a center atom. The algorithm starts with the initial atom identifier (in our case Daylight invariants [17]) of the center atom and grows a circular substructure around this atom. This growing can be done implicitly, like in the original algorithm, or explicitly, like in our variant. In the original algorithm the identifiers of the alpha atoms of a center atom are used to calculate an updated identifier for the center atom. In each iteration, the identifiers of the previous iteration are used as atom identifiers. This iterative procedure implicitly grows a circular substructure around the center atom because with an increasing number of iterations the updated identifier of a center atom contains information from further and further away. Our variant does the growing of a substructure explicitly by keeping the circular substructures of the previous iteration and their possible attachment points in memory. In each iteration the circular substructures are extended at their possible attachment points using the initial atom identifiers (Figure 1). Both ECFP variants generate an ECFP feature for each possible center atom and iteration. We evaluated the performance of both ECFP variants and could not observe a significant difference. We employed our variant because the information contained in a feature is defined more precisely.

To be able to look up the substructure information of an ECFP feature, we saved the mapping from fingerprint feature identifiers to circular substructures while calculating the fingerprints of a data set. Due to the hashing, which is conducted to assign a fingerprint feature identifier, it is possible that a features identifier maps to several different substructures. However, collisions can be minimized by choosing a sufficiently large hash space.

### Heat map molecule coloring

To allow a medicinal chemist to interpret a linear model, it is intuitive to color each atom or bond of a molecule according to its importance for activity. This coloring is achieved by the heat map molecule coloring.

Another intuitive approach to interpret a linear model is to select all patterns that exceed a certain weight threshold, as done in a study by Fechner et al. [8]. Their pattern selection approach proved to be useful on small data sets which share a common scaffold. However, preliminary experiments showed that the approach does not lead to interpretable results on large, diverse chemical data sets of assay outcomes, especially for external predictions. The top 5 ranked patterns (examples for several employed data sets can be found in Additional file 1) mostly had a significantly higher precision than expected by chance. However, the chance to find a significantly predictive pattern drops considerably with decreasing rank. Even for data sets with a promising performance (*AUC *> 0.9), the chance to find a singular predictive pattern in the set of selected patterns (weight ≥ 3 × *σ*) for the training set is small (*p *< 0.3). This probability drops considerably for the test set. A reason for this low probability might be that several patterns that by themselves cannot separate the two classes well might separate the classes better if combined [18]. For example, two patterns can perform perfectly random on their own, but can perfectly separate both classes if combined. However, combining patterns can not be accomplished by a method that inspects single patterns separately. Another reason can be the redundancy of the ECFP. The SVM might split the importance (weight) of a substructure among the redundant patterns that represent it.

Consequently, the patterns appear less important than they actually are. This problem is circumvented by the heat map coloring technique because it integrates the information of all training patterns. Hence, the importance of all redundant patterns that represent a certain substructure is fused in the coloring of a molecule. Additionally, in a chemical compound, patterns with different weights might overlap, making the interpretation unintuitive. In contrast, the information of overlapping patterns is integrated and intuitively visualized by the heat map coloring approach.

The inputs of our method are a previously trained linear SVM model, a list of compounds of interest and possibly the whole training data set depending on the employed normalization. Our method can be divided into two separate steps. First, our algorithm assigns a score to each atom and bond of a molecule based on the weights of the linear SVM model. Second, the scores are transformed to a color on a color gradient.

**Figure 2:**
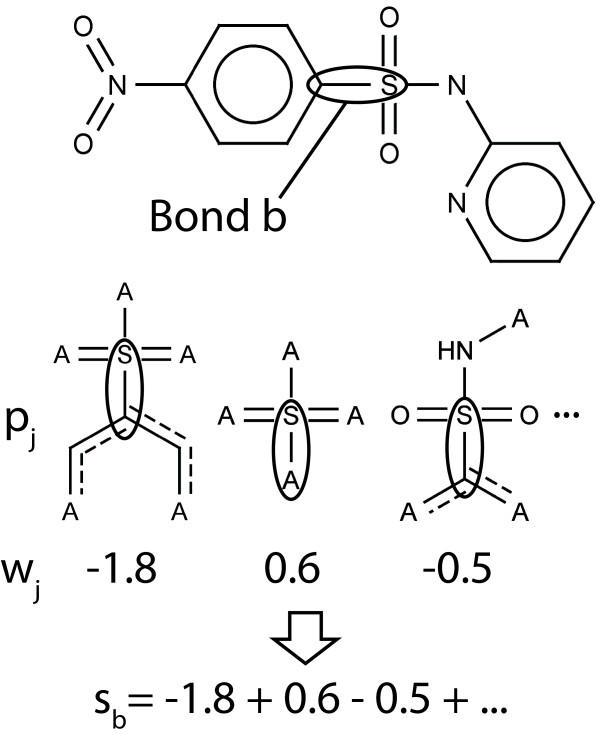
**Illustration of pattern to bond weight mapping**. The weight *w_j _*of a pattern *p_j _*is added to the score *s_b _*of a bond *b *if the bond is contained in the pattern *p_j_*. Attachment points A can be mapped on any atom. Aromatic bonds are marked by a dashed line.

In this manuscript, we only performed bond coloring. Thus, we only explain the calculation of bond scores. The calculation of scores is analogous for atoms and bonds. Hence, the presented calculation of bond scores can be easily transferred to atoms if needed. We had two reasons for solely using bond coloring. Firstly, the ECFP focuses on connectivity information which can be better visualized using bond coloring. Secondly, we wanted to allow for easy element identification by using element type atom coloring. For a different fingerprinting algorithm, like radial atom environments, atom colorings might be more useful. To assign a score to each bond, our algorithm fingerprints the compounds of interest or the whole training data set again. Throughout the fingerprinting process the information, which bonds a fingerprint feature represents in a certain compound, is stored. Based on the weights of the fingerprint features, a score is assigned to each bond of a compound. The score of a bond *b *is equal to the sum of weights of the fingerprint features that contain the bond (Figure 2). Thus, the score *s_b _*of a bond *b *is(4)

where *B*(*f*) is the set of bonds a feature *f *represents and *w*(*f*) is the weight of a feature *f *as found in the linear SVM model. In the case of ECFP features, the bonds to attachment points are included in the set of bonds a feature represents. Now, each bond of a processed compound has a score according to the weights of a trained linear SVM model. Therefore, the SVM model is responsible for assigning sensible weights if a smaller sub-fragment occurs in larger activating and non-activating fragments.

The score of a bond can not be transformed to a color directly. The score needs to be normalized to [0,1], which is achieved by(5)

where *s_max _*and *s_min _*are the maximum and minimum score found, respectively. Two different normalizations are possible depending on how *s_max _*and *s_min _*are chosen. The first method (full set normalization) chooses *s_max _*and *s_min _*to be the maximum and minimum score found throughout the fingerprint calculation on the whole training data set and the compounds of interest. The second approach (single molecule normalization) sets *s_max _*and *s_min _*to the maximum and minimum score found in the current compound.

Both normalizations have advantages and disadvantages. The full set normalization keeps the information of the prediction value differences between compounds. Generally, a compound with a larger prediction value has larger atom and bond scores. The relative differences of scores between compounds are not changed by the full set normalization. Thus, the relative differences in their prediction value are kept. A disadvantage of the full set normalization is that small differences between scores of a compound might not be visible. If the difference between two scores of a compound is small compared to the maximum score of the whole data set, then the difference is even smaller after normalization. Furthermore, the whole training data set needs to be fingerprinted again, which can take a considerable amount of computation time for large data sets. In contrast to the full set normalization, the single molecule normalization visualizes small differences in the scores of a compound better. Furthermore, the computation time is fast because only the compounds of interest need to be processed. The main disadvantage of the single molecule normalization is that the coloring does not contain any information about the overall activity because only differences within the molecule are taken into account.

Another normalization aspect concerns the calculation of the bond score *s_b_*. In our implementation we do not take the fragment size of a fingerprint feature into account. To take the fragment size into account, the contribution of a fragment to a bond score *s_b _*is divided by the number of bonds in the fragment. Thus, the weight of a feature is equally distributed among the bonds associated with the feature. This weight distribution lowers the influence of large fragments on the bond score *s_b_*. However, putting the focus on smaller fragments might be less suited for large, diverse HTS data sets, where the scaffold is most important. Additionally, experiments (not shown) indicated that in case of the ECFP, the colorings are smoother without equal distribution of weights. Thus, we decided not to use equal weight distribution for our visualization.

After normalization to [0,1], a score can be transformed to a color on a color gradient. We use a color gradient from red over orange to green, where red represents the negative class and green represents the positive class. The whole gradient can be divided into two sub-gradients, one from red to orange and another one from orange to green. The first sub-gradient is used if , the second one if . The two sub-gradients result in a smooth gradient from red to green when combined. Each sub-gradient is realized by mixing each color channel (R, G, B) of the two sub-gradient colors according to the normalized bond score :(6)

where *R_r_*, *R_o_*, and *R_g _*are the values of the R color channel of the respective gradient colors red, orange, and green. The colors are mixed for each color channel separately and the resulting color (*R_mix_,G_mix_,B_mix_*) is assigned to the respective bond.

## Experimental

### Virtual screening data sets

We conducted evaluation experiments on 17 maximum unbiased validation (MUV) data sets, an Ames toxicity data set (Kazius), and a chromosome aberration (CA) data set. A detailed analysis was conducted for the Kazius data set and two of the MUV data sets.

First, we employed the 17 MUV data sets compiled by Rohrer et al. [11] with their corresponding background data sets. Each of these data sets comprises 30 dissimilar active compounds together with 15,000 inactive compounds, which are similar to the actives with respect to several simple descriptors like volume, solubility, or mass. The MUV data sets are designed to avoid artificially high screening performance by inappropriate decoys. Additionally, the common spread of actives can have a positive impact on the interpretability of a model because the chance of overfitting a model on a small cluster of similar actives is minimized. Two of these data sets, MUV548 and MUV846, were subjected to a detailed analysis. MUV548 contains inhibitors of protein kinase A as actives and MUV846 contains inhibitors against factor XIa.

Second, we used the mutagenicity data set composed by Kazius et al. [10]. It comprises 2401 mutagenic and 1936 non-mutagenic compounds based on the Ames toxicity test. Kazius et al. derived 29 toxicophores from the data. Using these toxicophores, they could predict the toxicity of an external test set with an accuracy of 85% which is close to the theoretical limit of the Ames toxicity test. The authors provide a list of well defined toxicophores and demonstrate their predictive power on several compounds.

Third, we employed the CA data set compiled by Mohr et al. [6]. This data set consists of 351 positive and 589 negative compounds with respect to the chromosome aberration test. Mohr et al. achieved an accuracy of 89.5% on 10 predefined cross-validation folds. The data set was included because the authors' method provides visual structure-activity information on several compounds of the data set.

### Experimental setup

All data sets were prepared according to the guidelines by Fourches et al. [19]. The structures were canonicalized and transformed with JChem Standardizer [20]. The options of Standardizer were set to neutralize, tautomerize, aromatize, calculate clean 2 D coordinates, and add explicit hydrogens. Explicit hydrogens were added because CDK [21], the core library of the employed fingerprinting algorithms, requires correctly attached hydrogens bonded to an atom. Then, all employed data sets were checked for duplicates and fingerprinted using the ECFP variant with a depth of four and a hash space size of 2^22 ^to minimize collisions.

To evaluate the performance on each data set, we used a 5-fold two-deep cross-validation [22] which was repeated two times. We employed the large-scale linear SVM LIBLINEAR [23] to train a linear SVM model. On the CA data set we also performed an evaluation on the 10 defined splits of Mohr et al. The SVM parameter *C *and the weight of the negative class *W*_-1 _were searched using a 2-fold cross-validation.

For *C *we chose the grid *log*_2_(*C*) ∈ {-5, -4, ..., 7, 8} and for *W*_-1 _we used the grid *log*_2_(*W*_-1_) ∈ {-4, -2, 0}. We also discarded uncommon features from the data before building the model. A feature had to occur at least 3 times to be included in the training. For the detailed heat map coloring analysis we used the whole data set to train a model and only left the compound of interest for analysis out. The same *C *and *W*_-1 _grids were used.

We employed two different measures to evaluate the performance on the data sets. First, the accuracy (ACC) was computed, which is the number of correctly predicted compounds divided by the total number of compounds. The accuracy is only applicable for balanced data sets like the Kazius and CA data set. Second, we employed the area under the ROC curve (AUC). The ROC curve plots the fraction of correctly predicted actives (true positive rate) against the fraction of inactives incorrectly predicted as actives (false positive rate) for every possible threshold. The AUC is applicable for all used data sets. The higher the value of both measures, the better is the performance.

All employed PDB structures were prepared with the protein preparation wizard of Schrödinger 2010 [24]. The settings of the preparation wizard were set to the default settings.

## Results and Discussion

The results of the analysis of the 19 employed data sets are organized as follows. First, we present the performance on the employed data sets. Then, we briefly explain, why we selected MUV548, MUV846, and the Kazius data set for a detailed analysis and visualization. Finally, we demonstrate and discuss the heat map molecule coloring method on those three selected data sets.

### Selection of data sets for visualization

We selected the three data sets of the detailed analysis by two criteria. First, the performance of a linear model trained on a data set must be reasonably good because the predictive performance of a model should be crucial to obtain sensible structure-activity relationships. Second, to be able to validate the results of a visualization, literature information on structure-activity relationships of the target of a data set must be available.

The linear SVM could predict the CA data set on the 10 predefined splits with an accuracy of 72% (Table 1), which is comparable to the nonlinear SVM performance reported by Mohr et al. Although the performance of the linear SVM is considerably worse than the method of Mohr et al. (89,5%), we chose to further analyze this data set because Mohr et al. provide visualizations of several compounds of the CA data set. We predicted all compounds that were illustrated in their study externally. All of them were either predicted wrong or had a prediction value close to zero and thus were not convincing predictions. The heat map coloring showed few overlap with the substructures identified by their visualization method, which is presumably due to the 17% lower accuracy and therefore more inaccurate model. Hence, the predictive performance of a model seems to be crucial to obtain sensible structure-activity relationships with our method.

**Table 1:** Performance of LIBLINEAR on data sets

Data set	Target Class	AUC	ACC
CA	Genotoxicity	0.765	0.724
Kazius	Toxicity	0.912	0.842

MUV466	GPCR	0.644	-
MUV548	Kinase	0.900	-
MUV600	Nuclear Receptor	0.685	-
MUV644	Kinase	0.893	-
MUV652	RNAse	0.782	-
MUV689	Kinase	0.865	-
MUV692	Nuclear Receptor	0.584	-
MUV712	Chaperone	0.863	-
MUV713	PPI	0.784	-
MUV733	PPI	0.634	-
MUV737	PPI	0.687	-
MUV810	Kinase	0.822	-
MUV832	Protease	0.960	-
MUV846	Protease	0.958	-
MUV852	Protease	0.852	-
MUV858	GPCR	0.669	-
MUV859	GPCR	0.595	-

AUC and ACC performance of LIBLINEAR on the MUV, Kazius, and chromosome aberration data sets.

On the Kazius data set, the linear SVM achieved an accuracy of 84%, which is close to the theoretical limit of the Ames toxicity test. The convincing performance and the availability of defined toxicophores from Kazius et al. make this data set an ideal choice for a more detailed analysis.

The performance on the different MUV data sets varied between an AUC of 0.58-0.96. All data sets with kinase, protease and chaperone targets showed a promising AUC performance (0.82-0.96), whereas the protein-protein interaction and reporter gene dependent assays had a considerably worse AUC performance (0.58-0.78). It would have been interesting to analyze the data sets with a GPCR target because it is hard to get crystal structures for those targets. Therefore, information on structure-activity relationships can not be obtained from structure based modeling for GPCR targets, which would make information from our visualization valuable. However, the performance on these data sets is close to random, and thus, a visualization would not be sensible. We chose the MUV548 which can be predicted with an AUC of 0.90 and the MUV846 with an AUC of 0.958 for a detailed analysis and visualization with the heat map coloring method. The linear models of both data sets exhibit a top ranked performance compared to all data sets and plenty of literature is available for the protein targets. While the MUV832 has the best performance (0.96), there are no crystal structures, which contain a ligand similar to the data set, available. Therefore, the MUV832 was not subjected to a detailed analysis.

### Visualization of Kazius Ames toxicity data set

**Figure 3:**
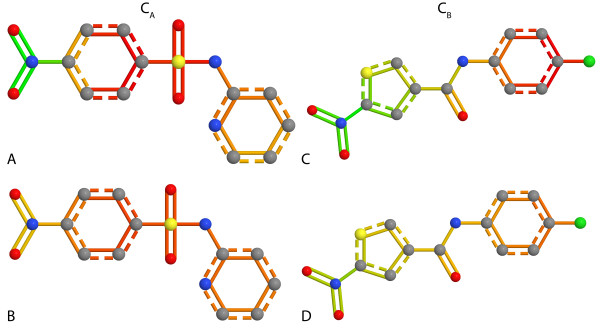
**Kazius data set example compounds**. A heat map coloring of the non-toxic compound 1028-11-1 (C*_A_*) and the toxic compound 146795-38-2 (C*_B_*). Both compounds were predicted correctly. The color gradient ranges from green (toxic) to red (non-toxic). Both, the single molecule normalization (A,C) and the full data set normalization were applied (B,D). Compound C*_A _*contains a correctly identified aromatic nitro toxicophore. However, the compound has a detoxifying sulfonamide as well, rendering the compound non-toxic. The sulfonamide and parts of the aromatic ring were identified as non-toxic. In compound C*_B _*the aromatic nitro toxicophore was also identified as toxicophore. Compound C*_B _*is toxic because the red chlorobenzene substructure is not a detoxifying substructure.

Using a linear model of the Kazius data set, we externally predicted compounds 1028-11-1 (C*_A_*) and 146795-38-2 (C*_B_*), and applied our heat map atom coloring method with both normalization variants. Figure 3(A,B) shows the heat map coloring of the correctly predicted non-toxic compound C*_A_*. The toxic and the detoxifying substructures described by Kazius et al. could be identified with our method. However, in addition to the detoxifying sulfonamide our method also colored parts of the aromatic ring structure red (non-toxic), which is probably caused by the fact that the sulfonamide is often attached to an aromatic ring in the data set. Compound C*_B _*(Figure 3C,D) was correctly predicted as toxic. The compound contains the same aromatic nitro toxicophore as compound C*_A_*, which our method identified together with parts of the attached aromatic ring. In contrast to C*_A_*, compound C*_B _*is toxic because it does not contain a detoxifying sulfonamide. However, the compound has a red colored chlorobenzene substructure, which is non-toxic and not detoxifying in case of C*_B_*. The overall toxicity of both compounds is visualized by the full set normalization. Compound C*_A _*(Figure 3B) is more reddish compared to compound C*_B _*(Figure 3D) and therefore has a lower prediction value.

The coloring of the compounds reveals weaknesses of both normalization methods. When the single molecule normalization is applied, one can not distinguish between a non-toxic and a detoxifying substructure because the normalization can only visualize differences within the structure. Thus, it is impossible to decide if a substructure is detoxifying or non-toxic without additional information on the toxicity. Given a compound that only contains toxicophores, the most toxic substructure would be colored green and the least toxic weighted substructure would be colored red. However, the information on the toxicity of a compound is available in form of the prediction value. Thus, this weakness can be compensated. The visualization of compound C*_A _*(Figure 3B) indicates the drawbacks of the full set normalization method: While it captures the overall toxicity of the compound, the aromatic nitro toxicophore and the detoxifying sulfonamide are less distinguishable.

### Visualization of MUV548 protein kinase A data set

We conducted external predictions for the ligands of the PDB entries 3MVJ (L*_A_*), 3DNE (L*_B_*), and 3DND (L*_C_*) using a model trained on MUV548. Then, we applied our heat map coloring to the ligands. The employed PDB entries were the most suitable ones of several crystal structures available for protein kinase A because of their similarity to the compounds contained in MUV548. The ligands of other PDB structures are more dissimilar and thus presumably not in the applicability domain of a model trained on MUV548. The crystal structures of L*_B _*and L*_C _*originate from a study by Orts et al [25]. The authors used an NMR based method to determine the binding orientation of low-affinity inhibitors. The method allowed for selecting the correct binding orientation of both ligands from four different orientations gained by rotation of the ligands in the binding pocket. To elucidate if our coloring method can also assist to select the correct binding orientation, we applied our method to the ligands presented in the study of Orts et al. and additionally ligand L*_A_*. The crystal structure of L*_A _*stems from an analysis of selective inhibitors against Akt1 (PKB) [26]. The ligand inhibits PKA with an *IC*_50 _of 3.2 *μM*.

**Figure 4:**
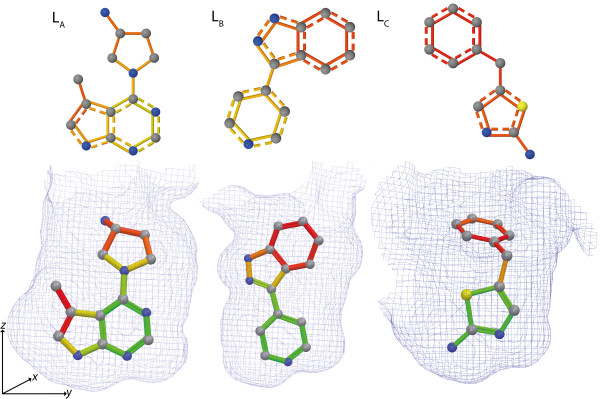
**Orientation of different protein kinase A ligands**. Binding orientation of the ligands of PDB entries 3MVJ (L*_A_*), 3DNE (L*_B_*), and 3DND (L*_C_*). Compounds within the binding pocket were colored with the single molecule normalization, the compounds above with the full set normalization. The color gradient ranges from green (important for activity) to red (unimportant or even decreasing for activity). The binding pocket is indicated as an exclusion surface. Substructures, which are located at similar positions in the binding pocket, were colored similarly by the heat map coloring approach.

The coloring of the substructures of all three ligands correlates with the substructure position in the binding pocket (Figure 4). Especially the position of the green colored basic aromatic ring deep in the binding pocket is conserved for the three structures. If the ligands were rotated by 180° around the *y *axis, the red colored (unimportant) substructures would be located deep in the binding pocket. Hence, our approach assists to find the correct orientation of the ligands if rotated around the *y *axis. This rotation excludes two of the possible four orientations described by Orts et al. However, in case of the presented ligands our approach can not help to discriminate between rotations around the *z *axis. Yet, this limitation is not a real drawback for our method because it can be applied on the data of an HTS without performing additional NMR experiments.

The external prediction value of ligand L*_C_*, as indicated by the full set coloring (Figure 4), is lower than the prediction value of the other two ligands. On the ranked list of prediction values the ligands L*_A _*and L*_B _*are under the top 1% while the ligand L*_C _*is at position 4, 206 of 15,003 compounds. Hence, the coloring of L*_C _*could be inaccurate and changed if the ligands are included in the training set. Thus, we added all three ligands to the training set and applied the heat map coloring again. As expected, the training prediction values of all three ligands then were under the top 1% of the ranked list of training prediction values. The new position of the ligand L*_C _*in the ranked list was reflected by the full set normalized coloring. However, the single molecule normalized coloring did not change considerably for any of the ligands. The change in the full set normalized coloring was caused by a positive weighting of a large substructure of ligand L*_C_*. Re-weighting large substructures does not substantially influence weight differences within the molecule. Hence, in the case of L*_C _*the single molecule normalization might be more robust than the full set normalization.

The approach to compare the colorings of an external prediction and an inclusion in the training set might be a way to estimate the robustness of a coloring. While test compounds should never be included in model training when building predictive models, our intend is to build a descriptive model to identify features crucial for a molecule's molecular behavior. In the later case, inclusion of a compound for model building might be beneficial because additional information for finding important features is available. However, if the coloring of a compound changes drastically after inclusion in the model training, the descriptive model might not be sensible or structural aspects of a certain scaffold were not included. A robust model should not swap from completely meaningless features to sensible features by inclusion of just one compound in model training.

**Figure 5:**
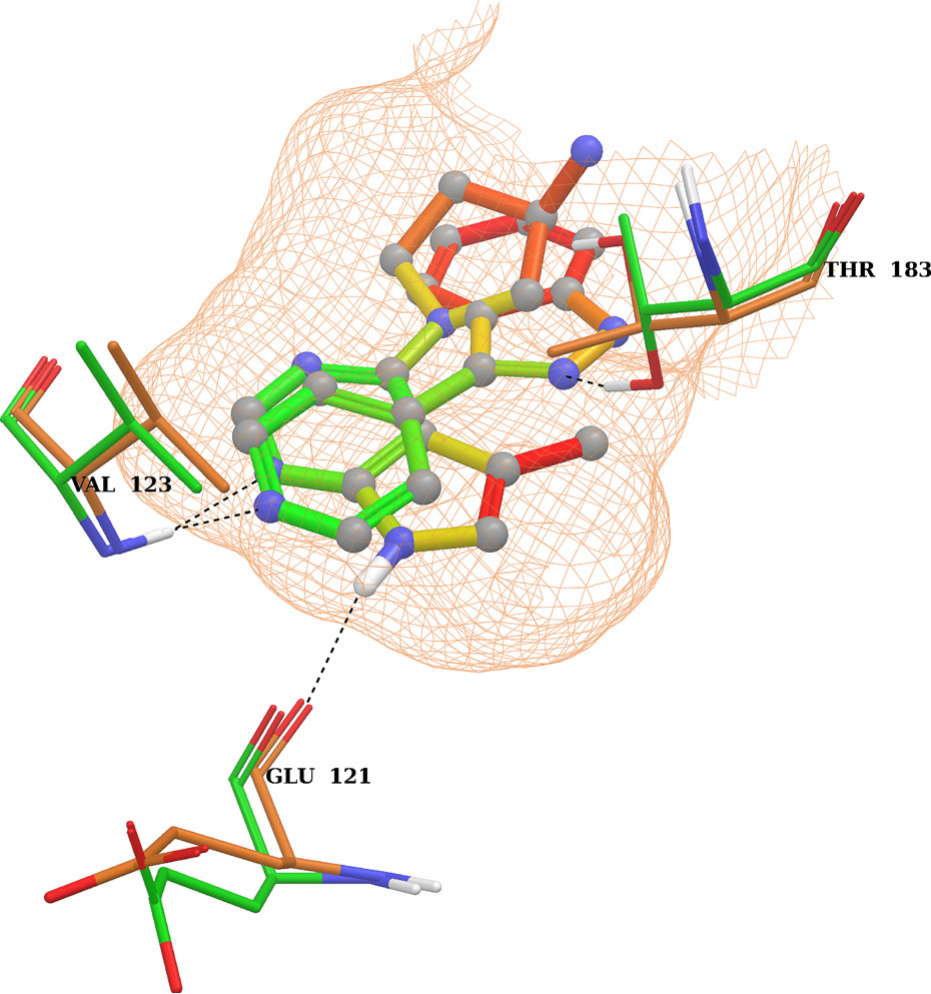
**Aligned binding pockets of L*_A _*and L*_B_***. The binding pockets of L*_A _*and L*_B _*were aligned and the ligands were colored with the single molecule normalization. The color gradient ranges from green (important for activity) to red (unimportant for activity or even decreasing). The green protein residues belong to L*_A _*and the orange ones to L*_B_*. The binding pocket is indicated as an exclusion surface. H-bonds detected by Schrödinger are indicated by a dashed line. Two similar basic aromatic rings located deep in the binding pocket are identified as important for activity.

To evaluate if our visualization colors those substructures important for the interaction between a ligand and the target, we aligned the binding pockets of the crystal structures of L*_A _*and L*_B _*using Schrödinger 2010 [24]. We chose L*_A _*and L*_B _*because the external prediction values of the ligands were within the top 1% of the ranked prediction value list. The important interactions of the ligands can be illustrated in comparison to the binding of ATP. The purine base of ATP is anchored in the binding pocket by hydrogen bonds to three protein residues: Glu121, Val123 and Thr183 [27,28]. In both compounds a basic aromatic ring substructure is marked as important for activity by the heat map coloring method (Figure 5). According to Schrödinger 2010, Val123 establishes an H-bond with the N1 of the pyridine of L*_B _*and the N1 of the pyrrolopyrimidine of L*_A_*. In the structure of L*_B_*, an additional H-bond connects Thr183 and the N1 of the indazole ring. In the structure of L*_A_*, Glu121 establishes an H-bond with the N7 of the pyrrolopyrimidine substructure. All H-bonds are reflected by the heat map coloring of the ligands. Additionally, the N1 of the pyrrolidine is colored as important for activity. While no interaction was detected for nitrogen N1, all active compounds of MUV548 that are based on a pyrrolopyrimidine scaffold also contain this nitrogen. Furthermore, parts of the pyrrolopyrimidine and the C5 attached methyl group of the ligand L*_A _*were marked as unimportant suggesting that the protein might be more flexible in this region. This flexibility assumption is supported by the form of the binding pocket of L*_C _*(Figure 4) which is not closed in the corresponding region. Consequently, the most important substructure, according to the heat map coloring approach, might be a basic aromatic ring substructure which is able to interact with Val123.

### Visualization of MUV846 factor XIa data set

As with protein kinase A (MUV548), a plethora of crystal structures with small compound ligands are available for factor XIa. We tested our approach on the 6 ligands of the PDB entries 1ZRK, 1ZSK, 1ZTL, 2FDA, 3BG8, and 1ZOM. We trained a model on MUV846 and conducted external predictions and heat map colorings on the ligands. All external predictions were ranked between position 3127 and 11762 on the ranked list of the prediction values of 15,006 compounds. We included all six compounds in the training set to estimate the robustness of the colorings, analogously to the setup for ligand L*_C _*on MUV548. All compounds, except the ligand of PDB entry 3BG8, had a considerably different coloring compared to the external prediction. Hence, in the case of these five ligands the colorings might not be robust and apparently do not reflect sensible structure-activity relationships.

**Figure 6:**
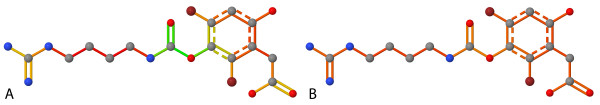
**Clavatadine A**. Clavatadine A colored according to a model trained on MUV846. The color gradient ranges from green (important for activity) to red (unimportant for activity). The current molecule normalization (A) and the full data set normalization (B) were both applied. The carbamate substructure is marked as important for activity.

PDB entry 3BG8 contains a natural product, Clavatadine A, which inhibits factor XIa with an *IC*_50 _of 1.3 *μM *[29]. Clavatadine A (Figure 6) is cleaved by a nucleophilic serine at the carbamate bond leaving only the carbamate side chain in the protein. Although the external prediction value of Clavatadine A is not convincing (position 3127 in the ranked list), the single molecule normalized heat map coloring of Clavatadine A identifies the carbamate bond as an important substructure for activity. A closer look at the active structures of MUV846 reveals that several active compounds also contain a carbamate bond and thus might exhibit the same binding mode. As with compound C*_A _*of the Kazius data set the full set normalization does not yield a useful coloring for Clavatadine A. The whole compound is colored reddish, which obscures the slight differences in coloring that are visible with the single molecule normalization.

## Conclusions

We presented a method to visualize structure-activity and structure-property information of a linear SVM model. The heat map coloring approach assigns a color to each atom or bond of a certain molecule according to the weights of a linear SVM model. The visualization combined with linear SVMs provide an information gain compared to black box machine learning approaches like nonlinear SVMs. The method does not only provide a prediction value to label a compound as active or inactive, but also provides reasons for the labeling. Although we only tested the visualization with linear SVMs, it should in principle not be limited to linear SVMs. The visualization only requires a machine learning algorithm to assign weights to molecular fingerprinting features. The benefit of combining the visualization with linear SVMs is their promising performance and fast computation time on large-scale data sets.

We introduced two different normalization schemes. The experiments revealed advantages and disadvantages of both normalizations. However, the single molecule normalization in combination with the prediction value might be the most valuable representation for the visualization of a compound.

We evaluated our approach on a toxicity data set, a chromosome aberration data set, and the MUV data sets. Overall, the experiments show that our method sensibly visualizes structure-property and structure-activity relationships of a linear SVM model. Thus, we conclude that our method can help to guide the modification of a compound in later stages of drug discovery.

On the Kazius data set, our method allowed for identification of the toxicophores of two example compounds and therefore might help in lead optimization to obtain a less toxic compound.

The results on the MUV data sets demonstrate that our method is able to determine the correct orientation of a compound in the binding pocket. Additionally, the heat map coloring allows for the identification of important substructures for ligand protein interactions or binding mechanisms without having protein structure information. Yet, it is impossible to elucidate the exact binding mechanism or interactions without structure based approaches. Thus, the heat map coloring should be considered as complementary to structure based approaches and as such help to get a better understanding of the binding mode of an inhibitor.

The approach is not suited for identifying important side groups of a common scaffold. This deficit is mainly caused by the diversity of the employed large-scale data sets. To allow the machine learning algorithm to focus on side groups, it is necessary to employ data sets in which all compounds share a common scaffold. However, those data sets are not in the scope of a classifier, but require regression techniques.

A focus in future studies might be the combination of heat map coloring with linear support vector regression in order to elucidate the contribution of side groups to activity or selectivity.

## Availability

All employed programs are available free of charge as executable jar and source code at http://www.ra.cs.uni-tuebingen.de/software/ChemHeatmap/. This includes the employed ECFP fingerprints, a modified Java version of LIBLINEAR and a graphical user interface to perform a heat map coloring of a compound. A short tutorial showing the workflow to obtain the colorings of the two compounds of the Kazius data set is also available.

## Competing interests

The authors declare that they have no competing interests.

## Authors' contributions

LR wrote the manuscript and implemented most of the code. GH and AJ assisted in the design and implementation of the fingerprinting algorithm. AZ supervised the study and participated in the discussion of the results. All authors read and approved the final manuscript.

## Supplementary Material

Additional file 1 **Number of support vectors and top weighted fragments**. For each data set with an *AUC *≥ 0.7 the number of support vectors and the top five weighted fragments are listed in the file. Click here for file

## References

[B1] Bajorath J Integration of virtual and high-throughput screening Nat Rev Drug Discov 2002 1 882 894 10.1038/nrd941 12415248

[B2] Bleicher KH Böhm HJ Müller K Alanine AI Hit and lead generation: beyond high-throughput screening Nat Rev Drug Discov 2003 2 369 378 10.1038/nrd1086 12750740

[B3] Bender A Mussa HY Glen RC Reiling S Molecular similarity searching using atom environments, information-based feature selection, and a naïve Bayesian classifier J Chem Inf Comput Sci 2004 44 170 178 14741025 10.1021/ci034207y

[B4] Han L Wang Y Bryant S Developing and validating predictive decision tree models from mining chemical structural fingerprints and high-throughput screening data in PubChem BMC Bioinformatics 2008 9 401 10.1186/1471-2105-9-401 18817552 PMC2572623

[B5] Swamidass SJ Azencott CA Lin TW Gramajo H Tsai SC Baldi P Influence relevance voting: an accurate and interpretable virtual high throughput screening method J Chem Inf Model 2009 49 756 766 10.1021/ci8004379 19391629 PMC2750043

[B6] Mohr J Jain B Sutter A Laak AT Steger-Hartmann T Heinrich N Obermayer K A maximum common subgraph kernel method for predicting the chromosome aberration test J Chem Inf Model 2010 50 1821 1838 10.1021/ci900367j 20883013

[B7] Hinselmann G Rosenbaum L Jahn A Fechner N Ostermann C Zell A Large-Scale Learning of Structure-Activity Relationships Using a Linear Support Vector Machine and Problem-specific Metrics J Chem Inf Model 2011 51 2 203 213 10.1021/ci100073w 21207929

[B8] Fechner N Hinselmann G Jahn A Rosenbaum L Zell A A Free-Wilson-like Approach to Analyze QSAR Models Based on Graph Decomposition Kernels Mol Inf 2010 29 491 497 10.1002/minf.201000053 27463327

[B9] Hasegawa K Keiya M Funatsu K Visualization of Molecular Selectivity and Structure Generation for Selective Dopamine Inhibitors Mol Inf 2010 29 793 800 10.1002/minf.201000096 27464269

[B10] Kazius J McGuire R Bursi R Derivation and validation of toxicophores for mutagenicity prediction J Med Chem 2005 48 312 320 10.1021/jm040835a 15634026

[B11] Rohrer SG Baumann K Maximum unbiased validation (MUV) data sets for virtual screening based on PubChem bioactivity data J Chem Inf Model 2009 49 169 184 10.1021/ci8002649 19434821

[B12] Free SM Wilson JW A mathematical contribution to structure-activity studies J Med Chem 1964 7 395 399 10.1021/jm00334a001 14221113

[B13] Kubinyi H Free Wilson Analysis. Theory, Applications and its Relationship to Hansch Analysis Quant Struct-Act Relat 1988 7 121 133 10.1002/qsar.19880070303

[B14] Schölkopf B Smola AJ Learning with kernels 2001 Cambridge, MA, USA: MIT Press

[B15] Ralaivola L Swamidass SJ Saigo H Baldi P Graph kernels for chemical informatics Neural Netw 2005 18 1093 1110 10.1016/j.neunet.2005.07.009 16157471

[B16] Rogers D Hahn M Extended-Connectivity Fingerprints J Chem Inf Model 2010 50 742 754 10.1021/ci100050t 20426451

[B17] Weininger D Weininger A Weininger JL SMILES. 2. Algorithm for generation of unique SMILES notation J Chem Inf Comput Sci 1989 29 97 101

[B18] Guyon I Elisseeff A An introduction to variable and feature selection J Mach Learn Res 2003 3 1157 1182 10.1162/153244303322753616

[B19] Fourches D Muratov E Tropsha A Trust, but verify: on the importance of chemical structure curation in cheminformatics and QSAR modeling research J Chem Inf Model 2010 50 1189 1204 10.1021/ci100176x 20572635 PMC2989419

[B20] ChemAxon JChem 5.3.8 http://www.chemaxon.com

[B21] Steinbeck C Han Y Kuhn S Horlacher O Luttmann E Willighagen E The Chemistry Development Kit (CDK): an open-source Java library for Chemo- and Bioinformatics J Chem Inf Comput Sci 2003 43 493 500 12653513 10.1021/ci025584y PMC4901983

[B22] Jonathan P Krzanowski WJ McCarthy WV On the use of cross-validation to assess performance in multivariate prediction Stat Comput 2000 10 209 229 10.1023/A:1008987426876

[B23] Fan RE Chang KW Hsieh CJ Wang XR Lin CJ LIBLINEAR: A Library for Large Linear Classification J Mach Learn Res 2008 9 1871 1874

[B24] Schrödinger LLC, New York, NY Schrödinger 2010 http://www.schrodinger.com

[B25] Orts J Tuma J Reese M Grimm SK Monecke P Bartoschek S Schiffer A Wendt KU Griesinger C Carlomagno T Crystallography-independent determination of ligand binding modes Angew Chem Int Ed Engl 2008 47 7736 7740 10.1002/anie.200801792 18767090

[B26] Freeman-Cook KD Autry C Borzillo G Gordon D Barbacci-Tobin E Bernardo V Briere D Clark T Corbett M Jakubczak J Kakar S Knauth E Lippa B Luzzio MJ Mansour M Martinelli G Marx M Nelson K Pandit J Rajamohan F Robinson S Subramanyam C Wei L Wythes M Morris J Design of selective, ATP-competitive inhibitors of Akt J Med Chem 2010 53 4615 4622 10.1021/jm1003842 20481595

[B27] Bossemeyer D Engh RA Kinzel V Ponstingl H Huber R Phosphotransferase and substrate binding mechanism of the cAMP-dependent protein kinase catalytic subunit from porcine heart as deduced from the 2.0 A structure of the complex with Mn2+ adenylyl imidodiphosphate and inhibitor peptide PKI(5-24) EMBO J 1993 12 849 859 8384554 10.1002/j.1460-2075.1993.tb05725.x PMC413283

[B28] Prade L Engh RA Girod A Kinzel V Huber R Bossemeyer D Staurosporine-induced conformational changes of cAMP-dependent protein kinase catalytic subunit explain inhibitory potential Structure 1997 5 1627 1637 10.1016/S0969-2126(97)00310-9 9438863

[B29] Buchanan MS Carroll AR Wessling D Jobling M Avery VM Davis RA Feng Y Xue Y Oster L Fex T Deinum J Hooper JNA Quinn RJ Clavatadine A, a natural product with selective recognition and irreversible inhibition of factor XIa J Med Chem 2008 51 3583 3587 10.1021/jm800314b 18510371

