# Supplementary material for: Interpreting linear support vector machine models with heat map molecule coloring
Source: J Cheminform. 2011 Mar 25;3:11. doi: 10.1186/1758-2946-3-11 (PMC3076244; doi:10.1186/1758-2946-3-11)
Supplement: Additional file 1 — Number of support vectors and top weighted fragments. For each data set with an AUC ≥ 0.7 the number of support vectors and the top five weighted fragments are listed in the file. [file 1758-2946-3-11-S1.PDF]

Example Fragments

The table shows additional information for data sets with an *AUC* ≥ 0.7. For each shown data set we report the number of support vectors, the fraction of features with weight 0, the AUC performance, and the five fragments with the largest weight. For each fragment we report its weight of, the number of its occurrences in the data set, and its classification precision.
The fragments were generated during the fingerprinting process by the CDK SMILES generator. The Daylight invariants assign a flag to an atom if it is contained in at least one ring. This flag is not encoded by the SMILES. Thus we flagged the atoms with an "(R)" if ring membership is not clear from the context. The ring can either be aromatic or non-aromatic. Thus, the type of the attached bonds might be unknown. The bond is drawn as a dashed line if the type of such a bond is unknown. If two fragments are depicted, the ECFP could not distinguish between the fragments or a collision occurred. Precisions are shown in **bold** if the precision is significantly higher than expected by chance. It is important to test the significance of a precision because it correlates with the number of occurrences for the MUV data sets. The correlation is due to the fact that the MUV data sets only contain 30 actives.

Data set	Number of SVs	Fraction of Zero Weights	AUC
Kazius	3313	0.026	0.912
**Fragments**	**Weight**	**Number of Occurences**	**Precision**
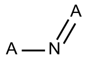	2.058	327	**0.789**
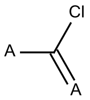	1.946	64	**0.875**
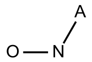	1.674	39	**0.923**
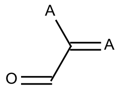	1.602	20	0.750
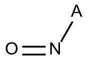	1.600	133	**0.895**
**Data set**	**Number of SVs**	**Fraction of Zero Weights**	**AUC**
CA	827	0.005	0.765
**Fragments**	**Weight**	**Number of Occurences**	**Precision**
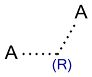	5.179	596	0.388
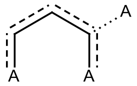	4.218	504	0.385
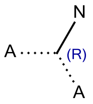	3.650	99	**0.566**
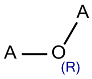	2.744	114	0.439
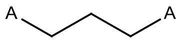	2.505	302	0.377

Data set	Number of SVs	Fraction of Zero Weights	AUC
MUV548	1105	0.700	0.900
**Fragments**	**Weight**	**Number of Occurences**	**Precision**
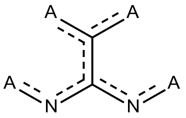	0.300	129	**0.078**
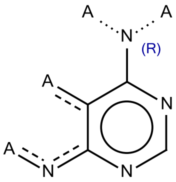	0.285	9	**0.556**
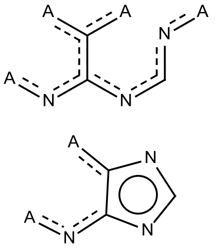	0.273	43	**0.209**
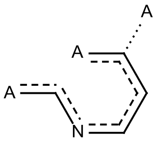	0.262	375	**0.035**
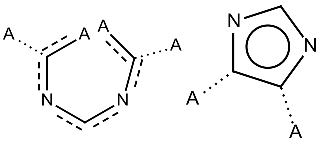	0.261	74	**0.135**

Data set	Number of SVs	Fraction of Zero Weights	AUC
MUV644	5370	0.267	0.893
**Fragments**	**Weight**	**Number of Occurences**	**Precision**
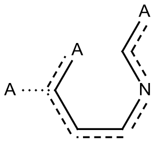	0.085	318	**0.041**
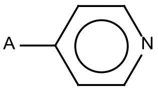	0.073	308	**0.039**
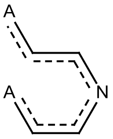	0.073	308	**0.039**
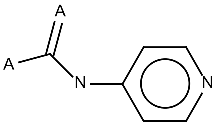	0.067	19	**0.263**
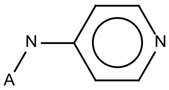	0.066	20	**0.250**

Data set	Number of SVs	Fraction of Zero Weights	AUC
MUV652	4225	0.312	0.782
Fragments	Weight	Number of Occurences	Precision
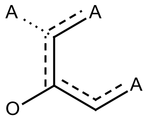	0.124	182	**0.044**
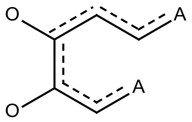	0.092	10	**0.400**
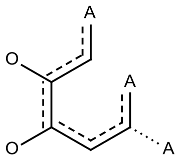	0.092	10	**0.400**
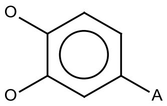	0.092	10	**0.400**
A=S	0.084	241	0.021
Data set	Number of SVs	Fraction of Zero Weights	AUC
MUV689	1883	0.603	0.865
Fragments	Weight	Number of Occurences	Precision
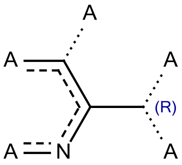	0.478	396	0.015
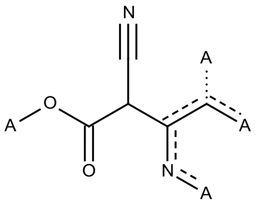	0.446	10	**0.300**
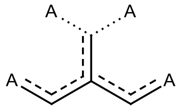	0.442	882	0.008
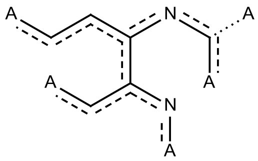	0.440	129	**0.039**
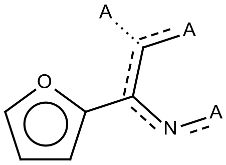	0.425	13	**0.231**

Data set	Number of SVs	Fraction of Zero Weights	AUC
MUV712	2354	0.537	0.863
**Fragments**	**Weight**	**Number of Occurences**	**Precision**
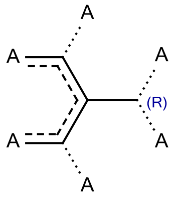	0.946	219	**0.037**
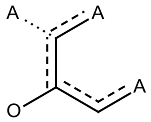	0.780	317	**0.038**
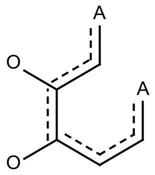	0.735	11	**0.363**
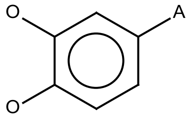	0.735	11	**0.363**
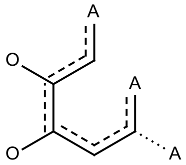	0.726	12	**0.333**

Data set	Number of SVs	Fraction of Zero Weights	AUC
MUV713	6713	0.168	0.784
**Fragments**	**Weight**	**Number of Occurences**	**Precision**
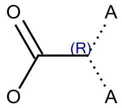	0.039	331	0.016
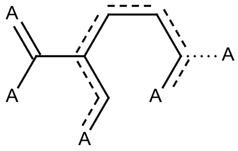	0.035	1529	0.006
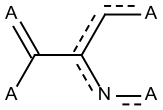	0.032	226	0.013
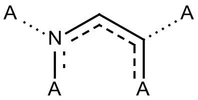	0.031	374	0.011
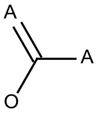	0.030	755	0.008

Data set	Number of SVs	Fraction of Zero Weights	AUC
MUV810	2851	0.476	0.822
**Fragments**	**Weight**	**Number of Occurences**	**Precision**
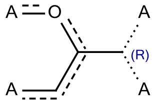	0.104	304	**0.013**
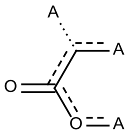	0.100	113	**0.027**
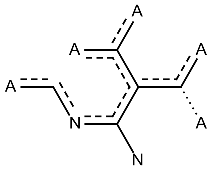	0.100	5	**0.400**
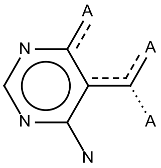	0.100	5	**0.400**
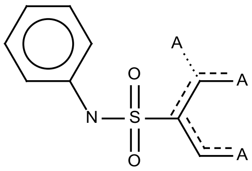	0.100	5	**0.400**
**Data set**	**Number of SVs**	**Fraction of Zero Weights**	**AUC**
MUV832	1566	0.648	0.960
**Fragments**	**Weight**	**Number of Occurences**	**Precision**
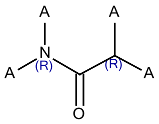	0.475	740	**0.015**
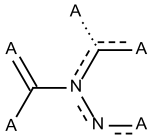	0.333	54	**0.130**
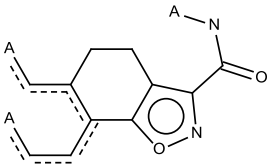	0.300	5	**1.000**
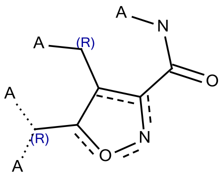	0.300	5	**1.000**

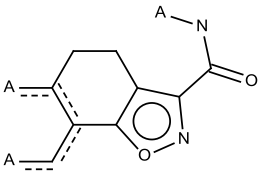	0.300	5	**1.000**
**Data set**	**Number of SVs**	**Fraction of Zero Weights**	**AUC**
MUV846	711	0.796	0.958
**Fragments**	**Weight**	**Number of Occurences**	**Precision**
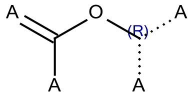	0.599	272	**0.051**
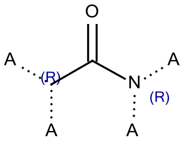	0.499	521	**0.015**
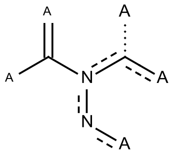	0.482	51	**0.118**
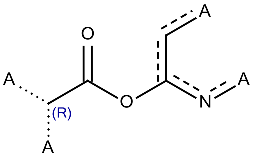	0.457	8	**0.750**
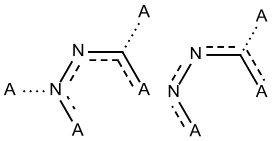	0.413	757	**0.015**
**Data set**	**Number of SVs**	**Fraction of Zero Weights**	**AUC**
MUV852	3753	0.396	0.852
**Fragments**	**Weight**	**Number of Occurences**	**Precision**
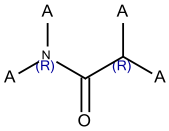	0.422	589	**0.029**
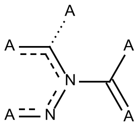	0.392	73	**0.178**
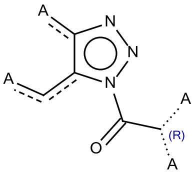	0.279	8	**0.875**
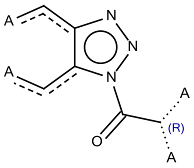	0.279	8	**0.875**

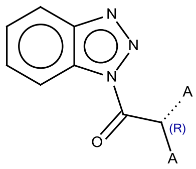	0.279	8	**0.875**
